# Improving Automated Annotation of Benthic Survey Images Using Wide-band Fluorescence

**DOI:** 10.1038/srep23166

**Published:** 2016-03-29

**Authors:** Oscar Beijbom, Tali Treibitz, David I. Kline, Gal Eyal, Adi Khen, Benjamin Neal, Yossi Loya, B. Greg Mitchell, David Kriegman

**Affiliations:** 1 Department of Computer Science and Engineering, University of California, San Diego, CA 92093, USA; 2 Charney School of Marine Sciences, University of Haifa, Haifa 3498838, Israel; 3 Integrative Oceanography Division, University of California, San Diego, CA 92093, USA; 4 The Interuniversity Institute for Marine Sciences in Eilat, Eilat 8810368, Israel; 5 Department of Zoology, George S. Wise Faculty of Life Sciences, Tel Aviv University, Tel Aviv 6997801, Israel; 6 Catlin Seaview Survey, Global Change Institute, The University of Queensland, Brisbane, Qld 4072, Australia

## Abstract

Large-scale imaging techniques are used increasingly for ecological surveys. However, manual analysis can be prohibitively expensive, creating a bottleneck between collected images and desired data-products. This bottleneck is particularly severe for benthic surveys, where millions of images are obtained each year. Recent automated annotation methods may provide a solution, but reflectance images do not always contain sufficient information for adequate classification accuracy. In this work, the FluorIS, a low-cost modified consumer camera, was used to capture wide-band wide-field-of-view fluorescence images during a field deployment in Eilat, Israel. The fluorescence images were registered with standard reflectance images, and an automated annotation method based on convolutional neural networks was developed. Our results demonstrate a 22% reduction of classification error-rate when using both images types compared to only using reflectance images. The improvements were large, in particular, for coral reef genera *Platygyra*, *Acropora* and *Millepora*, where classification recall improved by 38%, 33%, and 41%, respectively. We conclude that convolutional neural networks can be used to combine reflectance and fluorescence imagery in order to significantly improve automated annotation accuracy and reduce the manual annotation bottleneck.

Advances in robotics, control theory, and digital imaging technology have enabled the collection of large-scale digital photographic data-sets for a large variety of ecological surveys^1^,^2^,^3^,^4^. However, obtaining the relevant scientific data from the collected images typically requires time-consuming and expensive manual image annotation. Although recent automated annotation methods offer a compelling alternative to manual annotation^5^,^6^, the accuracy is rarely as high as that of human experts^7^,^8^,^9^. In this work we investigate whether multi-modal image channels can contribute to improved automated annotation accuracy, thereby reducing the need for manual image annotation and verification. Specifically, we focus on using fluorescence information, as a mode distinct from reflectance, for automated annotation of coral reef survey images.

Coral reefs are essential to coastal societies throughout the world, providing food, resources and income to over 500 million people^10^. In the last three decades up to 80% of coral coverage has been lost in the Caribbean^11^ and up to 50% in the Indo-Pacific^12^,^13^ largely due to anthropogenic stressors including over-fishing, pollution, sedimentation, habitat destruction and climate change^14^,^15^,^16^. This accelerated rate of decline creates a need for rapid assessments of reef health in order to develop more effective management and conservation strategies^17^.

Currently, the most prevalent method for reef assessment is *in situ* digital photographic surveys. However, obtaining ecological data, such as percent cover of key benthic groups, from the collected images requires time-consuming and expensive manual image analysis, often in the form of point annotations^18^.

Recent advances in computer vision have enabled automated annotation of coral reef survey images^6^,^19^,^20^,^21^, offering a compelling alternative to manual annotation^9^. Unfortunately, automated annotation in this context is challenging for several reasons including: degradation of colors underwater, image distortions due to water turbidity, large variability of within-class appearance, and complex boundaries and juxtaposition of different categories. As shown in this work, however, these difficulties can be alleviated by incorporating fluorescence information in the automated annotation methods.

While reef surveys are commonly conducted using underwater digital consumer reflectance cameras, several other instruments have been utilized to study coral reef health. Multi-spectral cameras, underwater spectrometers, underwater radiometers and underwater fluorometers (diving PAMs) have been previously used to quantify physiological parameters of coral reef organisms^22^. Another option is to measure fluorescence signatures which have been shown to contain ecologically relevant information such as level of bleaching^23^, recruitment^22^, and physiological state^24^. However, until recently, fluorescence imaging systems were limited either by resolution^24^, spatial coverage of the measurements^25^,^26^ and/or ease of operation^24^,^25^,^27^.

We have recently developed the FluorIS (Fluorescence Imaging System)^22^, a consumer camera modified for increased sensitivity of near-infrared wavelengths. The FluorIS can be used for underwater fluorescence imaging of both the green (520–630 nm), and red (630–800 nm), wide-band components of the fluorescence spectra which correspond to the emission spectra of green fluorescence proteins (GFPs) and chlorophyll-a, respectively. (The blue camera channel is not used as it overlaps with the spectrum of the blue excitation source). Its relatively low price, ease of operation, and wide-field-of-view (0.5 × 0.7 m^2^) makes FluorIS a compelling tool for reef surveys. We hypothesized that the information captured by FluorIS could be used as auxiliary information to improve automated image annotation accuracy.

To test this hypothesis we used a custom-designed framer and both a standard SLR and FluorIS camera to capture registered fluorescence and reflectance image pairs during a nighttime reef survey in Eilat, Red Sea Israel (Fig. 1). The images were then annotated by coral ecology experts at 200 random point locations as one of the ten dominant taxonomic categories. Several sets of analysis were then performed. First, the discriminatory information in each image channel was compared in order to understand the relative importance of the spectral bands in both cameras. Second, a supervised automated annotation algorithm, based on Convolutional Neural Networks (CNN)^28^, was developed which utilized the joint textural and spectral information in the image pairs. While CNNs have recently shown remarkable progress on several important visual recognition tasks, such as image classification^29^, and segmentation^30^, it has not previously, to the best of our knowledge, been utilized for automated annotation of underwater images.

We show that our CNN-based method was able to utilize the additional information captured by FluorIS to significantly improve the annotation accuracy compared to methods which only utilized the reflectance images^6^. This demonstrates that (1) fluorescence signatures contain information that can be directly related to the taxonomic identity of the benthos, and that (2) such signatures can be captured during reef surveys in conjunction with reflectance information. In addition, improved automated annotation accuracy can enable more extensive spatial and temporal studies by reducing the need for costly manual annotation and verification^9^.

## Results

### Analysis of image intensities

Overall classification accuracy using only image intensities (colors) was similar for the reflectance channels (76.9 ± 1.2%, [mean ± SE, n = 70]), and the fluorescence channels (77.5 ± 1.4%), Fig. 2a. However, there were differences in what categories that were accurately identified. The reflectance image intensities mostly allowed for accurate discrimination between bare-substrate and ‘unknown’, while the fluorescence image intensities allowed for discrimination between bare-substrate and live coral genera (Fig. 3). Indeed, the accuracy of classifying coral vs. other using the fluorescence channels was 88.3 ± 0.7%, but only 83.1 ± 0.9% for the reflectance channels (Fig. 2b).

The single most informative color channel was the green channel from FluorIS , which achieved 77.2 ± 1.4% overall accuracy, mostly due to a 65% recall for the dominant coral general, *Faciidae* (Figs 2a and 3). In contrast, the red channel, which was the most informative among the reflectance channels, achieved 70.7 ± 1.4% overall accuracy, mostly due to 58% recall for the ‘unknown’ category (Figs 2a and 3).

The annotation accuracy using all five channels was higher than the reflectance or fluorescence alone, achieving 82.2 ± 1.2% overall accuracy and 91.0 ± 0.5% accuracy of classifying coral vs. other (Fig. 2).

### Automated annotation using convolutional neural networks

While the results in the previous section were useful to understand the discriminatory information associated with the image intensities (colors), a reliable automated annotation method needs also to take into account texture and context in order to make more accurate annotation decisions^6^.

The overall annotation accuracy of *f* ^REF^, the CNN which used the reflectance images only, was 87.8 ± 1.1% (mean ± SE, n = 70; Fig. 4). This was lower than the 90.5 ± 0.8% accuracy of *f* ^JOINT^, which used information from both images (p < 0.0001, n = 70). This accuracy increase is equivalent to an error-rate reduction by 2.7% (from 12.2% for *f* ^REF^ to 9.5% for *f* ^JOINT^), which is a 22% relative reduction in error-rate, meaning that approximately one in five annotation errors were corrected by incorporating fluorescence information. The increase in performance was particularly large for coral genera *Platygyra* and *Acropora*, and for the hydrozoan genus *Millepora*, where the classification recall increased 38%, 33%, and 41%, respectively, which is a relative increase of 512%, 100% and 86% respectively from *f* ^REF^ (Figs 4 and 5).

The overall accuracy of *f* ^FLR^ (85.5 ± 1.2%), which used the fluorescence images only, was lower than *f* ^REF^ (p = 0.0002, n = 70), but higher on several important benthic substrates: *Platygyra*, *Acropora*, and *Millepora* (Figs 4 and 5). In addition, for *Pocillopora*, and *Faviidae*, the *f* ^FLR^ network, while less accurate than *f* ^REF^, provided complementary information that the joint *f* ^JOINT^ classifier utilized to outperform *f* ^REF^ (Figs 4 and 5). These findings support our main hypothesis that fluorescent information can improve the accuracy of automated annotation methods.

### Training a five-channel network

While CNNs are commonly used to model color or gray scale images, a CNN can operate on any number of channels^31^. Since the reflectance and FluorIS images are registered, they can be viewed as five-channel images, which offers a compelling opportunity to train a five-channel network directly on all the information from the image-pairs.

We trained a CNN, *f*^ FIVE^ directly on the annotated images of the training-set, and evaluated the accuracy, as described previously, on the test-set. This method directly incorporates both image types without the need for a subsequent second-stage classifier. The accuracy of *f* ^FIVE^ was 88.9 ± 1.1% (mean ± SE, n = 70), which was higher than the 87.8 ± 1.1% accuracy of *f* ^REF^ (p < 0.0001), but lower than the 90.5 ± 0.8% accuracy of *f* ^JOINT^ (p = 0.0029, n = 70).

### Comparison to traditional automated annotation methods

This work is the first to utilize CNNs for automated annotation of benthic survey images. It was therefore critical to determine how the accuracy of CNNs relate to more traditional methods based on hand-tuned filter or color descriptors explicitly designed for automated annotation of reflectance images^6^,^19^,^20^,^21^. Such methods commonly report accuracies of around 70–80%^19^,^20^; however, this is highly dependent on the data-set on which they are evaluated^9^,^21^. For this reason, we used the toolbox of Beijbom *et al.*^6^, which is publicly available (vision.ucsd.edu/content/moorea-labeled-corals), as a representative for the ‘traditional methods’, to compare the efficacy of such methods against the CNNs used in this work. The method of Beijbom *et al.*^6^ encodes image color and texture using a pre-defined set of image filters, and then uses a Support Vector Machine to learn models for each class. This method, denoted 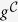 with the super-script indicating the image-type (REF or FLR), was trained and evaluated on the same training-set and test-set used throughout this work. The accuracy of *g* ^REF^ was 87.7 ± 0.7% (mean ± SE, n = 70), which was not different from the 87.8 ± 1.1% accuracy of *f* ^REF^ (p = 0.22, n = 70). When trained on the FluorIS images, the traditional method, *g* ^FLR^ achieved 81.0 ± 0.9% accuracy, which was lower than the 85.5 ± 1.2% accuracy achieved by the proposed CNN-based method, *f* ^FLR^ (p < 0.0001).

## Discussion

We have demonstrated that fluorescence information can be captured during an image-based benthic reef survey and utilized to improve the annotation accuracy of automated annotation methods. Our work is novel from three key perspectives. First, we used fluorescence information to improve the classification accuracy of the dominant benthic categories by 2.7%, which is equivalent to a 22% relative reduction of error-rate. In contrast to previous work that investigated the use of fluorescence spectra to classify groups defined by their level of bleaching^23^, fluorescence signatures^27^,^32^, or other physiological states^24^,^33^,^34^, our work provides evidence that the fluorescence signatures can be directly related to benthic taxonomy. Second, most work on coral fluorescence has relied on relatively expensive spectrometers or custom multi-spectral cameras which have high spectral but limited spatial resolution^27^,^32^. In contrast, we use an inexpensive, modified consumer camera, the FluorIS, which has high spatial resolution but only two spectral channels. The high spatial resolution and sensitivity of FluorIS enables deployment alongside a reflectance camera for regular benthic surveys. Third, we are the first, to the best of our knowledge, to use CNNs for automated annotation of joint fluorescence and reflectance image data. We believe this type of method can be applied to other ecological applications deploying multi-spectral or multi-modal cameras, e.g. vegetation monitoring^35^, inter-tidal landscapes^36^, or plant health for agronomic applications^37^.

The utility of the fluorescent information depended on the substrate class. Different coral species have different types of fluorescence proteins (FPs) and some species are more fluorescent than others^22^,^38^,^39^. In our study the fluorescence data was most helpful for improving the classification accuracy of the highly fluorescent coral genera *Platygyra* and *Acropora*, as well as for the hydrozoan *Millepora*. For these genera, the classification recall improved by 38%, 33% and 41% respectively (Fig. 4). We expect that including fluorescent information will increase the automated annotation accuracy of most coral reef surveys in areas with a large number of highly fluorescence species and in benthic surveys with a variety of fluorescent organisms (e.g. various algae species, diverse corals and other invertebrates). We also hypothesize that fluorescent information might assist in distinguishing between different types of algae that can be challenging to distinguish using automated annotation methods such as turf algae and CCA^9^; but additional studies will need to be performed in order to verify this hypothesis. Salih *et al.*^33^ carried out a survey of the distribution of fluorescent corals on the Great Barrier Reef and found that 124 species from 56 genera contained fluorescent morphs, suggesting that fluorescence should be a highly successful factor for improving annotation accuracy on coral reefs. However, they also found that within a given species there were often fluorescent and non-fluorescent morphs growing side by side. This fluorescence polymorphism within a coral species could explain why the fluorescence information did not improve the annotation accuracy further, and raises interesting ecological questions not explored here. Salih *et al.*^33^ also found that the highest number of fluorescent morphs occurred at the shallowest sites, and suggested that fluorescent pigments could have photo-protective properties. On the other hand, widespread, bright, and spectrally diverse coral fluorescence in meso-photic habitats^40^ may imply other properties of fluorescence pigments. Future work should explore the utility of fluorescence information for improving automated annotations at different depths, in different reef locations, and across different benthic habitats.

**Figure 1:**
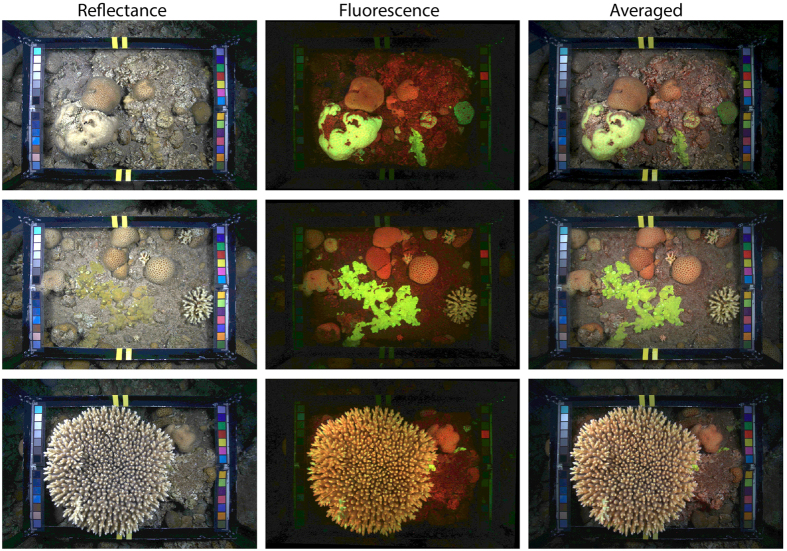
Three registered image pairs. (Left) Reflectance images, (Center) FluorIS images. (Right) Pixel-wise average of the reflectance and fluorescence images, visually demonstrating the advantage of combining the two information sources. The fluorescence increases the contrast of the corals, and the reflectance gives context and information on the non-fluorescing substrates. The registration quality is evident from this average. A high resolution version of this figure is available as supplementary information.

**Figure 2:**
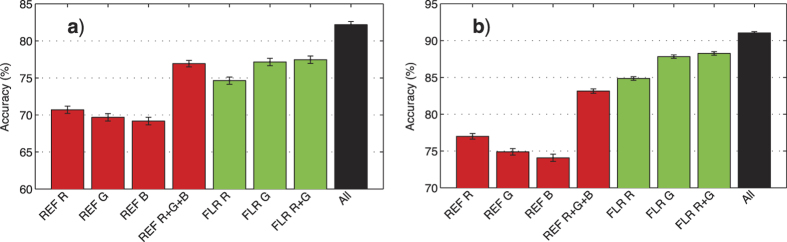
Classification accuracy based on image intensities. Results displayed as mean ± SE for (**a**) the full 10-category label-set and (**b**) coral vs. other. REF denotes the reflectance camera, FLR the fluorescence camera, and R, G, B denote the red, green and blue color channel respectively. The black rightmost bar indicate the accuracy of using FLR R+G together with REF R+G+B.

**Figure 3:**
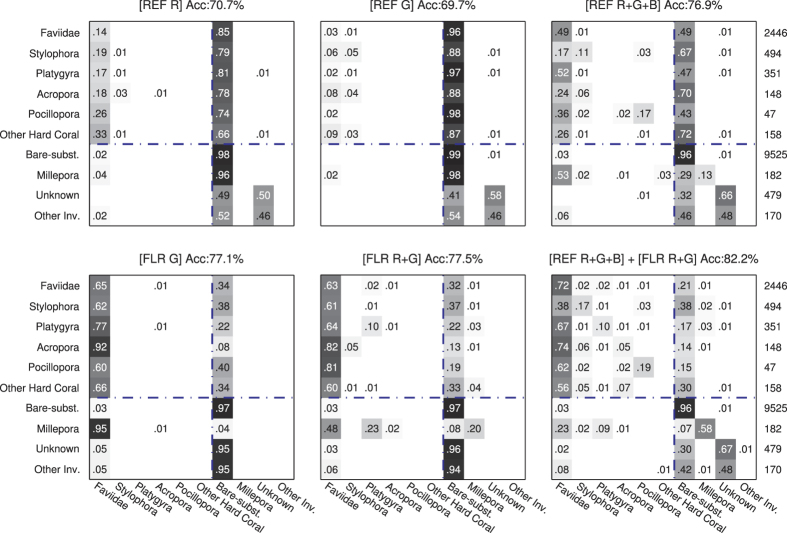
Confusion matrices for classification using image intensities. Numbers given on row r and column c are the probability of classifying category r as category c, and the total number of samples in each category is given on the right. The values along the diagonal indicate the recall of each category. REF denotes the reflectance camera, FLR the fluorescence camera, and R, G, B denote the red, green and blue color channel respectively. The blue dash-dotted lines separate the hard-corals from the other substrates.

**Figure 4:**
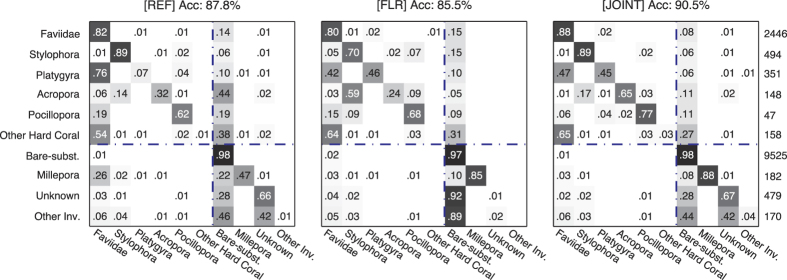
Confusion matrices for the proposed convolutional neural network method using information from (left) the reflectance camera, (middle) the FluorIS camera, and (right) both cameras. Numbers given on row r and column c are classification recalls, i.e. the probability of classifying category r as category c, and the total number of samples in each category is given on the right. The values along the diagonal indicate the recall of each category. The blue dash-dotted lines separate the hard-corals from the other substrates.

**Figure 5:**
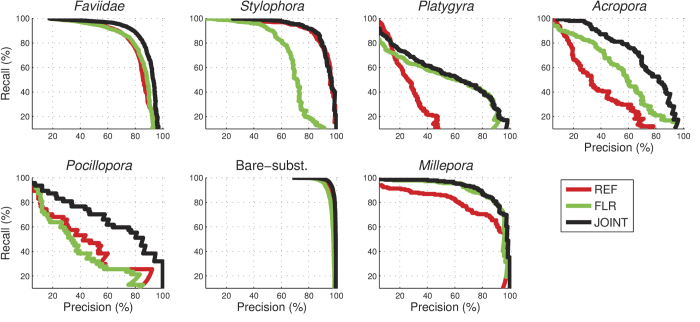
Precision vs. recall curves for *f* ^REF^, *f* ^FLR^, and *f* ^JOINT^. For each class, precision is defined as the proportion of correct classification among all samples classified as that class, and recall is defined as the proportion of correct classification among all samples from that class. The curves were created by varying the classification threshold for each class.

**Figure 6:**
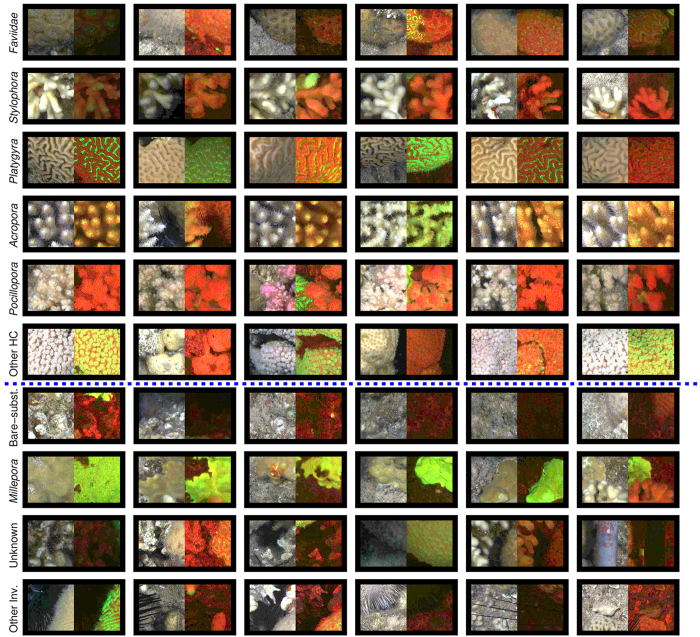
Cropped patch-pairs from the data set. Six randomly selected pairs of patches from each class are cropped out from the annotated point locations. The left member of each pair is from the conventional reflectance camera and right member is from FluorIS . The dotted blue line separates the hard corals from other substrates. A high resolution version of this figure is available as supplementary information.

Furthermore, our experiments have indicated the importance of careful registration of the image pairs. This is supported by the stronger results of the joint classifier, *f* ^JOINT^, which merged the information on the patch-level, compared to the five-channel network, *f* ^FIVE^, which merged the information on the pixel-level. We believe that this difference in accuracy was an effect of the registration quality. As shown in Figs. 1 and 6, the registration was good, but not perfect. Had it been perfect, with every pixel of the two images corresponding, we believe the *f* ^FIVE^ network would have done better, as it had direct access to all image information. Also, in our experiments, when the registration was omitted, the joint classifier failed to outperform the accuracy of the *f* ^REF^ network. This emphasizes the importance of careful registration and the development of new imaging systems and protocols for registered image capture.

Higher accuracy of an automated annotation system can reduce the amount of expensive human effort required during image annotation^9^. Our results show that a 22% reduction in error-rate can be achieved by incorporating fluorescence information, which means that one fifth of the automated annotation errors can be corrected if fluorescence information is available. This suggests that it may be cost-effective to deploy a wide-field-of-view fluorescence imaging system with a reflectance camera during surveys, in order to reduce the human annotation time. However, we emphasize that the image collection utilized in this survey should be considered a first attempt at obtaining co-registered images, and that additional work is required to facilitate robust co-located image capture of fluorescence and reflectance images on a large scale. Ideally, the image-pairs should be captured in a way that does not require a manual post-processing registration step. In addition, we previously showed that daytime fluorescence imaging is possible with the FlourIS using ambient light subtraction^22^. Future camera development that integrates reflectance and fluorescence channels in the same camera system together with automatic acquisition of an ambient light image would facilitate acquiring registered images at daytime.

We have also shown how to utilize CNNs for automated annotation of benthic survey images. To contextualize our results, we compared the efficacy of the CNNs against the method of Beijbom *et al.*^6^. This comparison showed that the CNNs performed on par with the method of Beijbom *et al.*^6^ on the reflectance images, but that it more effectively utilized the fluorescence information. These results were not surprising. Traditional automated annotation methods, such as that of Beijbom *et al.*^6^, were optimized on reflectance images, and could be expected to perform well on such images. However, the FluorIS images were of a different character both in terms of contrast and texture. For example, as we have shown, the FlourIS image intensities were more effective in discriminating between coral and non-corals than the reflectance image intensities. Therefore, traditional automated annotation methods, which were not designed for fluorescence imagery, perform poorly. CNNs, in contrast, were able to adapt and learn directly from this new type of data.

## Methods

### Image Collection

**Figure 7:**
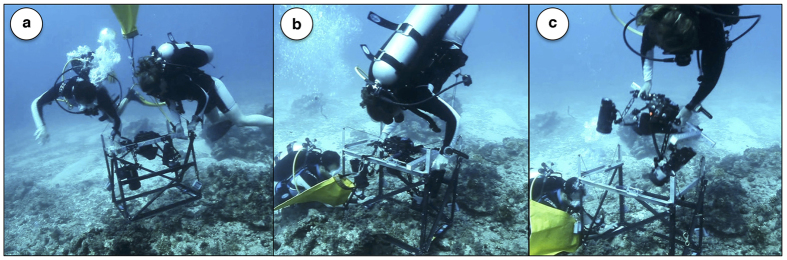
Image collection procedure. Two research divers first positioned a custom framer which has a docking system for the cameras (**a**). The FluorIS image was captured (**b**). The FluorIS camera with its four strobes was un-docked and removed from the framer (**c**). Finally, the reflectance camera was docked to the framer and a co-located reflectance image captured (Not shown). Using this procedure the two images have the same field-of-view and viewing angle.

Co-located image-pairs were captured during nighttime in the shallow reefs adjacent to the Interuniversity Institute for Marine Sciences in Eilat, Israel. Nighttime deployments were used due to the higher effectiveness of the fluorescence imaging system^22^ and the logistical ease of night diving in Eilat. Image locations were chosen randomly along a 3–15 m depth gradient. A custom-made framer was used that enabled rapid attachment and release of the imaging systems including the cameras and strobes (Fig. 7). To capture co-located image-pairs, the framer was carefully placed on the ocean floor and remained there while the cameras where docked, one after the other, so that the two images had the same field of view and viewing angle. Once both images were captured, the framer was moved to the next location. At each location, reflectance and fluorescent images were thus taken, each covering 50 × 70 cm of the benthos. Using this methodology, 212 image-pairs were collected during three dives, each approximately one hour long. The quality of the high-resolution images was sufficient for identifying most of the corals to the genus level, with some identification to the species level. All images and annotations used in this work is made publicly available at doi:10.5061/dryad.t4362.

For both reflectance and fluorescence imaging, the camera system comprised a Canon 5D Mark II professional grade off-the-shelf camera with a Sigma 20 mm wide-angle lens, and a Sea&Sea underwater housing with the Fisheye Dome Port 240, fitted with a 40 mm extension ring for better alignment of the dome port with the lens to reduce distortions. For fluorescence imaging, we used the Fluorescence Imaging System (FluorIS) which we developed in previous work^22^. In the FluorIS , the infrared filter on the camera sensor was removed for increased sensitivity to red fluorescence (chlorophyll-a emission). In addition, a yellow Tiffen #12 barrier filter was mounted on the camera lens, and blue NightSea excitation filters were mounted on the strobes as previously described^22^. As the modified FluorIS camera had an expanded spectral range in the long wavelengths, an additional filter (Schott BG39) was used for the strobes to block IR wavelengths that pass through the primary excitation filter. The fluorescence images were captured with camera settings at or around: 1/200 s, f/8.0, and ISO 800. Refer to^22^ for more details on the FluorIS including excitation spectra.

The reflectance camera system was deployed with two Ikelite DS161 (guide number 24) strobes while the FluorIS system used two Sea&Sea YS250s (guide number 32) and two Inon Z240s (guide number 24) to maximize the fluorescence signal. The reflectance images were captured with camera settings at or around: 1/200 s, f/8.0, and ISO 180.

### Post-processing

Using the custom framer, co-registered images were captured (Fig. 1). However, there were some minor registration issues due to slight framer movements. To ensure a high-quality registration, we therefore applied a post-processing registration step. In this step, four to ten corresponding points were hand-clicked in all image-pairs, and an affine image warp was applied using the imtransform command from the MATLAB Image Processing Toolbox (The MathWorks, Inc).

**Figure 8:**
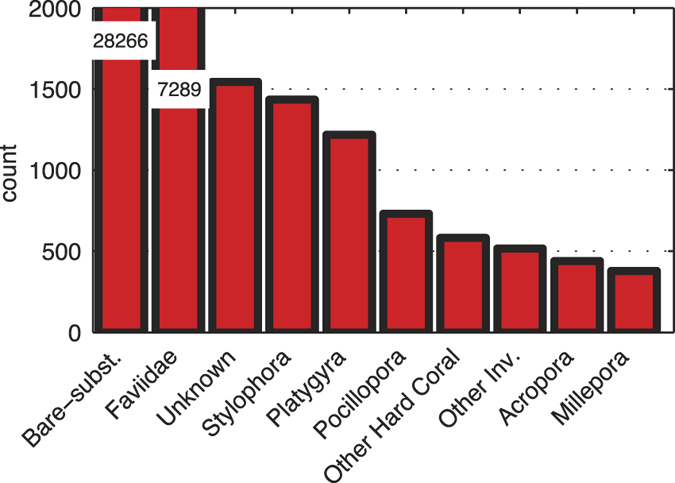
Histogram of point annotation counts across the 212 image-pairs. The y-axis is truncated at 2000 to ensure sufficient resolution for the rare labels, and the numbers overlaid the two leftmost bars indicate the actual height of those bars. The total number of annotated points is 42,400.

Image annotation of the reflectance images was performed by trained coral ecology experts (co-authors: AK, GE, and DIK) with knowledge of the local ecosystem. In this procedure, the substrate under 200 random point locations in each image was assigned one of ten pre-defined labels using the point-annotation tool of CoralNet (coralnet.ucsd.edu). The label-set included the five dominant coral genera: *Faviidae, Stylophora, Platygyra, Acropora* and *Pocillopora*; and non-coral labels for bare-substrate and colonial hydrozoans *Millepora*. The label-set also included ‘unknown’, which was used when identification was not possible (e.g. for dark image areas), as well as ‘other hard-coral’ and ‘other invertebrates’. A ‘macroalgae’ label was used during annotation, but was excluded from the subsequent analysis since it was exceedingly rare (<0.03%). The bare-substrate label included crustose coralline algae (CCA) and turf-algae since the distinction between these substrates was difficult, and sometimes not possible. A histogram of the number of annotations per category is shown in Fig. 8 and example patches from each of the labels are shown in Fig. 6. Since our goal was to evaluate the utility of fluorescence information for *automated* annotation, the “ground truth” was the human expert annotations of reflectance images, which is the standard used in previous studies^6^,^21^. Therefore, fluorescence images were *not* used during the manual annotation process. To verify the accuracy of the human expert, 1500 point annotations across 30 images were repeated by a second expert. A comparison of the two sets of manual annotations indicated excellent agreement (97.8%) between the two experts.

### Automated Annotation

For the purpose of method evaluation, 212 annotated image-pairs were divided into two sets. The training-set comprised 142 randomly selected image-pairs, and was used to train the automated annotation methods. The test-set comprised the remaining 70 image-pairs and was used to evaluate the annotation methods. With 200 point annotations per image, the training-set contained 28,400 point annotations, and the test-set contained 14,000.

### Discriminatory information in reflectance and fluorescence imagery

Before seeking to incorporate fluorescence information into an automated annotation system, we first wanted to verify that fluorescence image intensities indeed contain discriminatory information. Since benthic substrates lack distinct outlines, and since colors often change too much with the underwater conditions to be relied upon for classification^6^,^21^, automated annotation methods chiefly rely on textural cues^6^,^21^. On the other hand, fluorescent spectra have been shown to contain information that distinguishes between benthic substrates,^32^ which suggests that the colors (i.e. image intensities) of the fluorescence images can be used directly as predictive information in an automated annotation method. To determine whether fluorescence intensity provides useful discriminatory information, we conducted the following experiment.

Image intensities at the labeled locations were extracted from the training-set and used to train Support Vector Machine (SVM). We use a kernelized SVM with a Radial Basis Function kernel, as implemented in the LIBSVM software package^41^. A separate SVM was trained for each set of color channels, e.g. the red channel of the reflectance camera, or the red and green channels of the fluorescence camera. Hyper-parameters of the SVM were chosen by cross-validation on the training-set for each set of channels according to standard protocol^41^. The trained SVM was then used to predict labels on the images in the test-set. Finally, the predicted labels were compared to the ground-truth labels provided by the human expert in order to determine the classification accuracy for each set of image channels. Accuracy was evaluated both for the whole 10-category label-set and for a binary classification task: coral vs. other, where all hard-coral categories were merged to a generic ‘coral’ category and all remaining categories were merged to a generic ‘other’ category in post-processing.

### Automated annotation using convolutional neural networks

Our proposed automated annotation system used CNNs^28^. CNNs are commonly trained on color or gray-scale images, i.e., with one or three image channels^29^. The registered reflectance and FluorIS images collected in this work, however, have five channels of information (the FluorIS blue channel does not contain any information as it is filtered out^22^). We investigated two approaches for utilizing CNNs on this data. The first was to train a CNN directly using the five-channel information, and the second was to train two separate CNNs, one on each image type, and then train a second-stage classifier to consolidate the output of the two networks. In our experiments, the second approach was more effective and we focused our method development on this.

**Figure 9:**
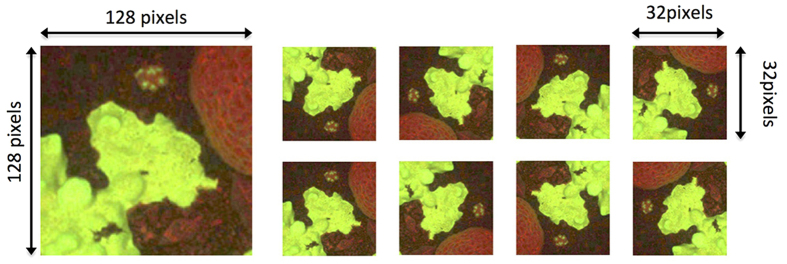
Training-set preprocessing procedure for the proposed automated annotation method based on convolutional neural networks. Around each annotation point-location, a 128 × 128 pixel patch was cropped (left). The patch was then resized using bilinear interpolation to 32 × 32 pixels, rotated (by 90, 180, and 270 degrees), and mirrored. This generated eight training-patches from each annotated point location (right).

We used Caffe^31^, an open-source framework for training the CNNs. Specifically, we used the publicly available cifar10 network structure (caffe.berkeleyvision.org), which is designed to learn from 32 × 32 pixels images. The cifar10 network comprises three rounds of consecutive convolutions, pooling and non-linear rectification layers, and all network parameters are learned directly from the training-set through back-propagation^42^. For each of the 200 annotated point locations in the training-set images, a centered 128 × 128 pixel patch was cropped out, and re-sized to 32 × 32 pixels. Rotated (by 0, 90, 180 and 270 degrees) and mirrored versions of each cropped patch were included in the training data in order to prevent over-fitting (Fig. 9)^29^,^30^. Using this data-preparation procedure, two networks were trained. Denoting 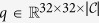 as an image patch with channels 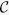, and by 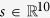 a vector of classification scores for each of the 10 categories in the label-set, the networks can be written as a mapping 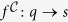. Classification of a new patch, *q*^∗^, from the test-set was done by assigning the class, *y*, corresponding to the largest score:


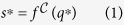



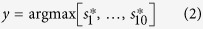


*f* ^REF^ represents the network trained on the reflectance images, and *f* ^FLR^ , the network trained on the FluorIS photos. To consolidate the information from the two networks a second-stage classifier was used that mapped the concatenated network scores to class labels. To create this second-stage classifier, all image patches were propagated through the networks after training. This generated two, 10-dimensional score vectors for each patch (one for each network and image type). The score vectors were then concatenated to a 20-dimensional joint score vector for each patch, which encoded information from both the reflectance and fluorescence images. The joint score vectors pertaining to the training-set were used to train a linear Support Vector Machine^43^, which was then used to predict the labels of the patches in the test-set. This second stage classifier was denoted *f* ^JOINT^, and is described in more detail below. Using a NVIDIA Tesla K40 GPU, the full network was trained in approximately 5 hours, and prediction took less than 1 second per image.

### Statistical analysis

A Wilcoxon signed-rank test was used to test the hypothesis that the annotation accuracy of *f* ^JOINT^ was higher than the *f* ^REF^ network. In other words, we examined whether the information from the FluorIS images improved annotation accuracy compared to using reflectance images alone. The accuracy was calculated for each image in the test-set as the ratio of correctly classified point-locations, where “correctly” is defined as agreeing with the human expert. Let *a*^JOINT^(*i*) be the accuracy of *f *^JOINT^ for image *i*, and *a*^(REF)^(*i*) the accuracy of *f*^REF^ for the same image. The paired annotation accuracy differences were then calculated as *d*(*i*) = *a*^JOINT^(*i*) − *a*^REF^(*i*), and *d* was used to perform the Wilcoxon signed-rank test. Following standard notation, we let *p* denotes the likelihood that the null hypothesis is true, and *n* the sample size. All tests were evaluated at the 95% confidence level, meaning that differences were considered significant for tests where p < 0.05.

## Additional Information

**How to cite this article**: Beijbom, O. *et al.* Improving Automated Annotation of Benthic Survey Images Using Wide-band Fluorescence. *Sci. Rep.* **6**, 23166; doi: 10.1038/srep23166 (2016).

## Supplementary Material

Supplementary Figure 1

Supplementary Figure 2
