# Supplementary figures and images for: Improving Automated Annotation of Benthic Survey Images Using Wide-band Fluorescence
Source: Sci Rep. 2016 Mar 29;6:23166. doi: 10.1038/srep23166 (PMC4810379; doi:10.1038/srep23166)

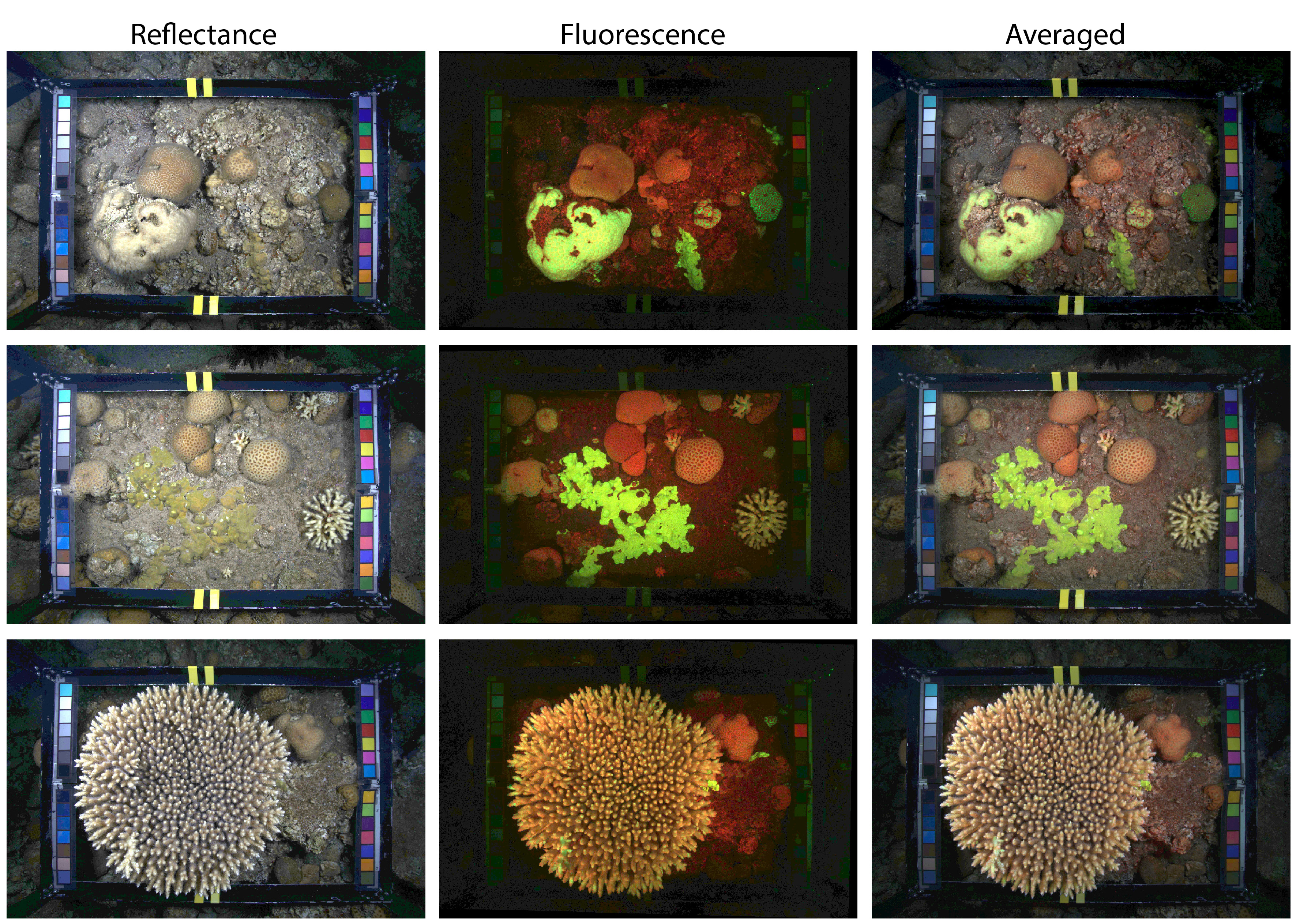

Supplement: Supplementary Figure 1 [file srep23166-s1.jpg]

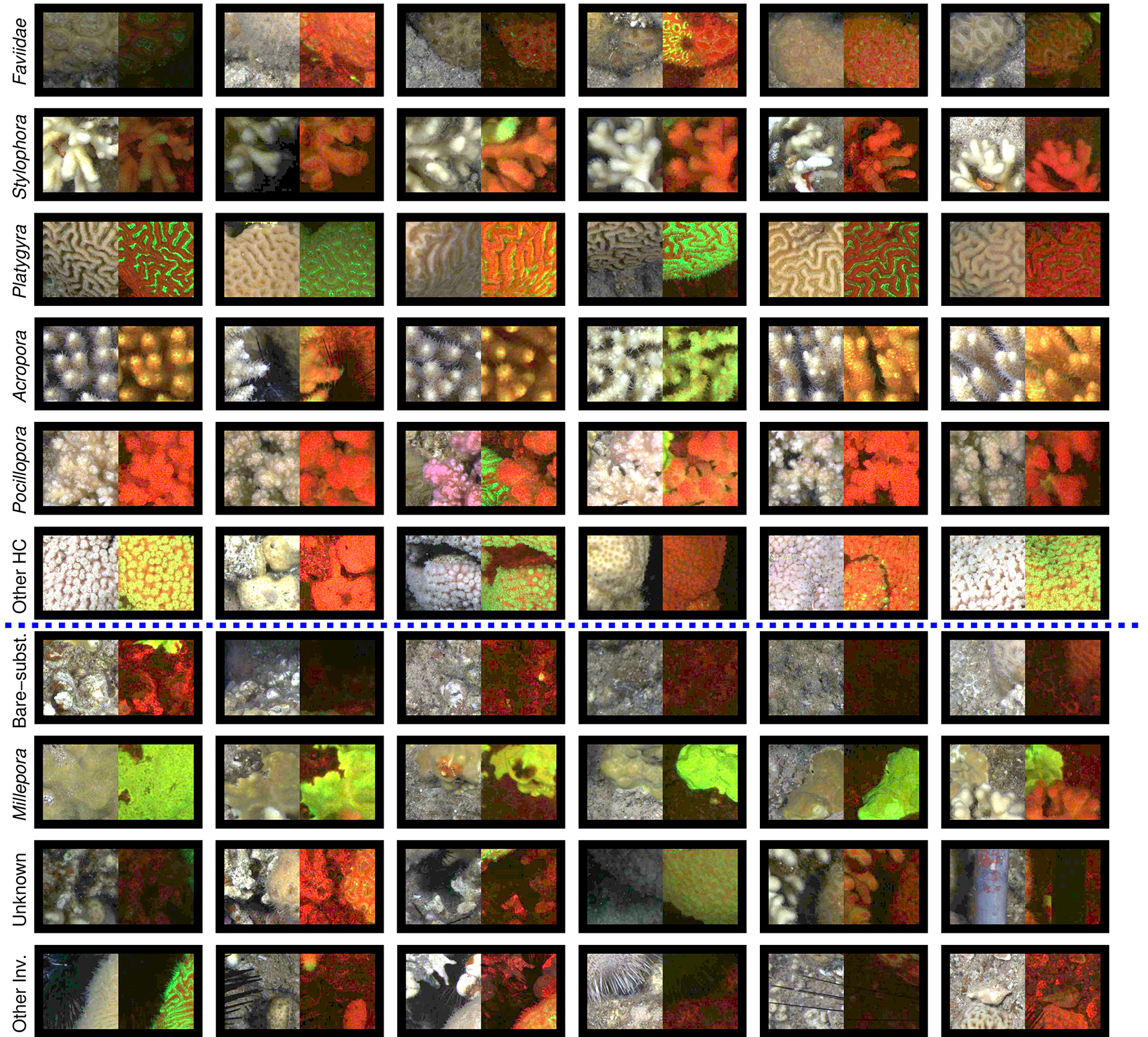

Supplement: Supplementary Figure 2 [file srep23166-s2.jpg]
